# Modeling and impedance matching for radio frequency driven plasma lamp considering cold and hot states

**DOI:** 10.1371/journal.pone.0203041

**Published:** 2018-09-18

**Authors:** Wonshil Kang, Hyunchul Ku

**Affiliations:** Department of Electronics Engineering, Konkuk University, Korea; University of Scranton, UNITED STATES

## Abstract

A new dual-state impedance matching scheme for a microwave driven plasma lamp using a solid-state power amplifier (SSPA) is presented. The impedance of the plasma lamp depends on the amount of input radio frequency (RF) energy, and therefore has very different values for hot and cold states. First, a method for effectively modeling the electrical characteristics of a plasma lamp that depends on RF power has been proposed. Second, a new technique has been proposed to achieve dual-state impedance matching for two state impedances at two very close frequencies using a T-shaped matching network with two section shunt stub and additional transmission line. The proposed method can achieve dual state impedance matching in two frequency bands located very closely when compared to the conventional methods. The accuracy of the proposed model and the effectiveness of the proposed dual-state matching are verified via a plasma lamp system with a 2.45 GHz 300 W GaN SSPA.

## Introduction

Electrodeless plasma lamps have excellent optical performance in terms of their sun-like spectrum, long lifetime, and high efficacy (lumens per watt) [1, 2]. Plasma lamps operate by supplying radio frequency (RF) energy (such as 450 MHz or 2450 MHz) to the lamp using a solid-state power amplifier (SSPA). The plasma lamp consists of a resonator and a bulb comprising materials such as Ar and InBr. The bulb has two different states: the off and on states, which correspond to the cold and hot states, respectively. The impedance of plasma lamps varies according to the RF energy that is supplied to the bulb [3, 4]. It is impossible to match two very different impedances simultaneously at one frequency. In result, we need additional ignition process (such as contacting metal ignitor in the bulb or applying very high voltage signal to the bulb) to turn on the plasma lamp if the output matching for the hot-state is only considered. To solve this problem, we propose an advanced matching technique for two different load impedances at two very close frequencies (the ratio of the two frequencies is about 1.005). This letter describes the extraction of an accurate equivalent circuit model of a plasma lamp with electrical characteristics that vary depending on the state. A new dual-state impedance matching design technique using a transmission line (T-line) based on the proposed model for the plasma lamp has been suggested, and its effectiveness has been verified with a plasma lamp system with a 300 W Gallium nitride (GaN) SSPA.

## Plasma lamp modeling

**Fig 1:**
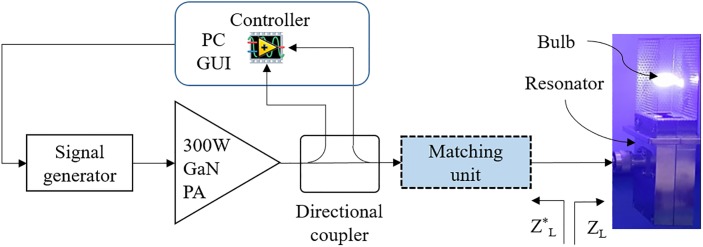
System block diagram of the electrodeless plasma lighting system.

**Fig 2:**
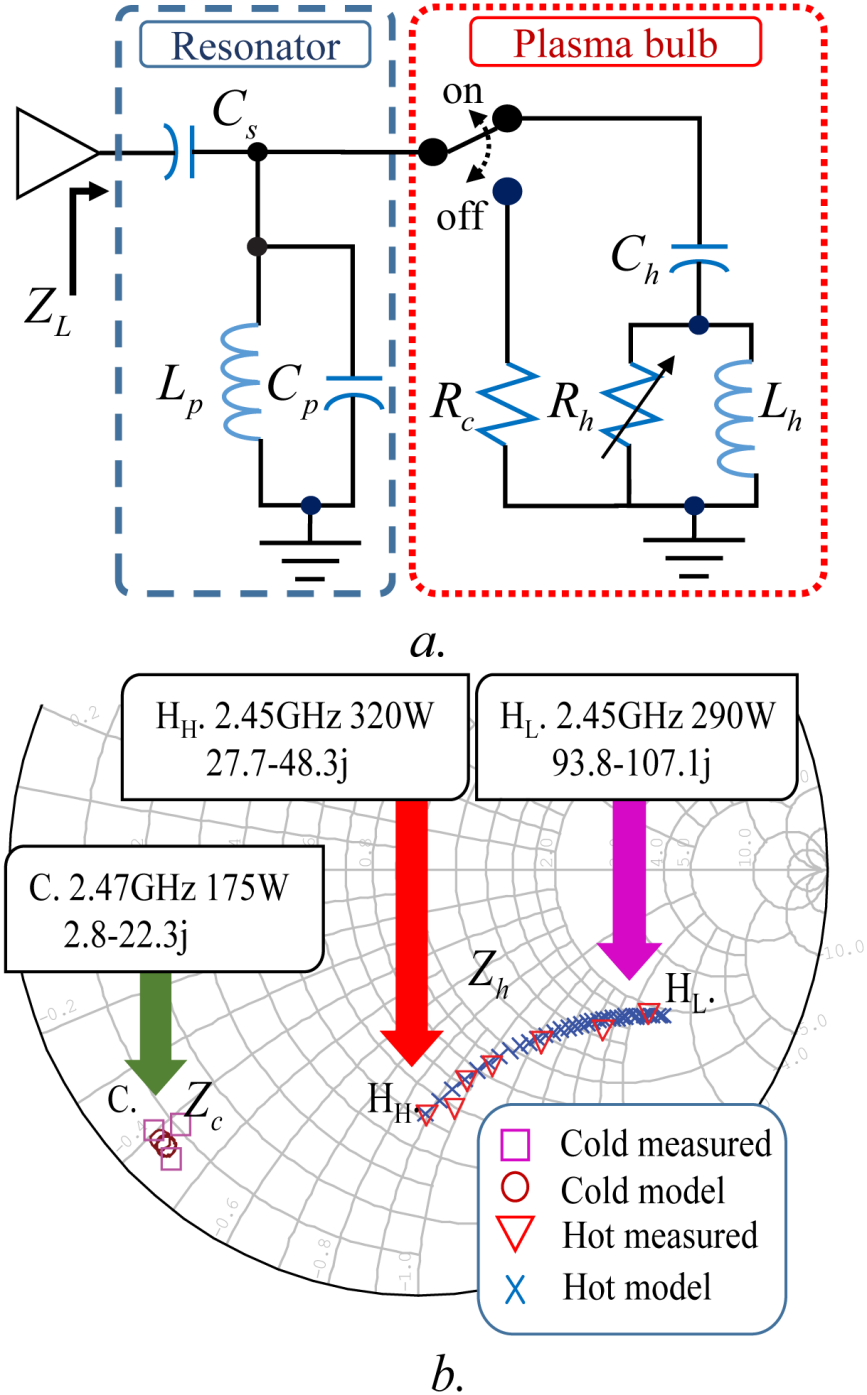
Electrical model of plasma lamp (Fig 2*a*), and comparison of measured and simulated impedances (Fig 2*b*).

An electrodeless plasma lighting system (PLS) using an RF SSPA consists of a signal generation component (NI USRP-2901), power amplifier (RFHIC MEL-500), matching component, resonator, plasma bulb (RFHIC URF-SP22), and a controller, as shown in Fig 1. The plasma lamp is comprised of a resonator and plasma bulb [2, 4, 9]. The impedance of the lamp is expressed as *Z*_*L*_ in Fig 2; the bulb impedances *Z*_*c*_ and *Z*_*h*_ respectively differ for the cold and hot states. In addition, the impedance of the hot state varies according to the RF energy supplied to the bulb [3, 4]. The impedance of the resonator with the plasma bulb is calculated by measuring the magnitude and phase difference of the reflected power using a directional coupler (ZGBDC35-93HP). A circuit model that incorporates both *Z*_*c*_ and *Z*_*h*_ is developed in consideration of the plasma lamp structure. *Z*_*h*_ has a different resonance frequency from *Z*_*c*_, and increasing the energy on hot-state reduces the resistance of the plasma bulb [5]. An equivalent circuit model of a plasma lamp is suggested in Fig 2*a*. The resonator in the lamp is modeled with series capacitor *C*_*s*_, shunt parallel inductor *L*_*p*_, and capacitor *C*_*p*_. *R*_*c*_ is used to represent the bulb in the cold state; the bulb in the hot-state is represented by implementing a variable resistor *R*_*h*_, inductor *L*_*h*_, and capacitor *C*_*h*_. The resulting impedances are as follows:

$$\begin{array}{c} Z_{h}=\frac{1}{j\omega C_{p}+\frac{1}{j\omega L_{p}}+\frac{j\omega C_{h}\left(R_{h}+j\omega L_{h}\right)}{R_{h}+j\omega L_{h}\left(j\omega C_{h}R_{h}+1\right)}}+\frac{1}{j\omega C_{s}},\\ Z_{c}=\frac{1}{j\omega C_{p}+\frac{1}{j\omega L_{p}}+\frac{1}{R_{c}}}+\frac{1}{j\omega C_{s}} \end{array}$$ (1)

The measured *Z*_*c*_ and *Z*_*h*_ are shown in Fig 2*b*. We measure the impedance of the plasma lamp in the off state (Fig 2*b*; Point C) and operating state (Fig 2*b*; Point H_L_ to H_H_). The parameters of the equivalent circuit model extracted from the measured data are shown in Table 1.

**Table 1:** Parameters of the plasma bulb model.

*C*_*s*_ [nF]	*C*_*p*_ [nF]	*L*_*p*_ [*μ*H]	*R*_*c*_ [Ω]	*C*_*h*_ [nF]	*L*_*h*_ [nH]	*R*_*h*_ [Ω]
3.02	0.441	6.19	3.2	3.11	38.65	34(H_H_)-226(H_L_)

A comparison of the data simulated using AWR ^®^ and the data obtained via measurements is presented in the form of a Smith chart (Fig 2*b*; S1 File). The impedances simulated via the proposed model are in good agreement with the measured impedances as shown in Fig 2*b*.

## Dual-state impedance transformer

**Fig 3:**
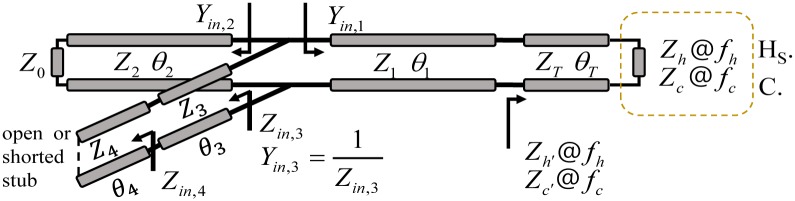
Proposed T-shaped dual-state matching design schematic.

Because the impedance of the plasma lamp in the hot-state is different from that in the cold-state, an impedance matching technique is required to reduce the cold- and hot-state return losses at frequencies of *f*_*c*_ and *f*_*h*_, respectively. The narrow gap of the two frequencies is advantageous for the PLS, because the frequency range in which SSPA typically operates with high efficiency is narrow. The best configuration of PLS is possible when the gaps of both frequencies are zero, but it can not be implemented. There are several techniques to perform dual-impedance matching [6–8], but it is difficult to derive design parameters satisfying a narrow frequency gap. To design an implementable impedance transformer, we propose a method using T-shaped T-lines with double-section shunt stubs, as shown in Fig 3.

*Z*_*n*_ (*n* = 1, 2, 3, 4) and *θ*_*n*_ are the characteristic impedances and electrical length of the T-line, respectively, for a frequency *f*_*h*_. The electrical length of the T-line is *mθ*_*n*_ for *f*_*c*_, where *m* = *f*_*c*_/*f*_*h*_. If the impedances of the plasma lamp are not within a certain range, T-lines with characteristic impedances *Z*_1_ and *Z*_2_ are difficult to implement, because the values of *Z*_1_ and *Z*_2_ are either too high or low. To overcome this problem, we change the impedance from *Z*_*h*_ and *Z*_*c*_ to *Z*_*h*′_ = *R*_*h*′_ + *jX*_*h*′_ and *Z*_*c*′_ = *R*_*c*′_ + *jX*_*c*′_, respectively, using series T-lines with characteristic impedance *Z*_*T*_ and electrical length *θ*_*T*_ for the frequency *f*_*h*_. *Z*_*h*′_ and *Z*_*c*′_ are determined as follows:

$$\begin{array}{c} Z_{h^{{}^{\prime }}}=Z_{T}\frac{Z_{h}+jZ_{T}\tan\theta_{T}}{Z_{T}+jZ_{h}\tan\theta_{T}},\;Z_{c^{{}^{\prime }}}=Z_{T}\frac{Z_{c}+jZ_{T}\tan\left(m\theta_{T}\right)}{Z_{T}+jZ_{c}\tan(m\theta_{T})}. \end{array}$$ (2)

Input admittances *Y*_*in*,*n*_(*f*)(*n* = 1, 2, 3, 4) at multiple ports are defined in Fig 3; *Y*_*in*,*n*_(*f*_*h*_) and *Y*_*in*,*n*_(*f*_*c*_) are the input admittances of the hot and cold states, respectively. Conditions to achieve impedance matching for *Z*_*h*′_ at a frequency of *f*_*h*_ and *Z*_*c*′_ at a frequency of *f*_*c*_ are as follows:

$$\begin{array}{c} Y_{in,1}\left(f_{h}\right)=Y_{in,1}^{*}\left(f_{c}\right),\;Y_{in,2}\left(f_{h}\right)=Y_{in,2}^{*}\left(f_{c}\right),\\ Y_{in,3}\left(f_{h}\right)=Y_{in,3}^{*}\left(f_{h}\right) \end{array}$$ (3)

where * denotes the conjugate of a complex value. The *Y*_*in*,1_(*f*_*h*_) and *Y*_*in*,1_(*f*_*c*_) are as follows.

$$\begin{array}{c} Y_{in,1}\left(f_{h}\right)=\frac{Z_{1}+j\left(R_{h^{{}^{\prime }}}+jX_{h^{{}^{\prime }}}\right)\text{tan}\theta_{1}}{Z_{1}\left(R_{h^{{}^{\prime }}}+jX_{h^{{}^{\prime }}}+jZ_{1}\text{tan}\theta_{1}\right)},\\ Y_{in,1}\left(f_{c}\right)=Z_{1}\frac{Z_{1}+j\left(R_{c^{{}^{\prime }}}+jX_{c^{{}^{\prime }}}\right)\text{tan}\left(m\theta_{1}\right)}{\left(R_{c^{{}^{\prime }}}+jX_{c^{{}^{\prime }}}+jZ_{1}\text{tan}\left(m\theta_{1}\right)\right)} \end{array}$$ (4)

The equation $Y_{in,1}\left(f_{h}\right)=Y_{in,1}^{*}\left(f_{c}\right)$ is divided into a real part and an imaginary part, and the equation tan(*a* + *b*) = (tan *a* + tan *b*)/(1 − tan *a* tan *b*) is applied to obtain the following equations.

$$\begin{array}{c} \tan(\theta_{1}+m\theta_{1})=\frac{Z_{1}(R_{h^{{}^{\prime }}}-R_{c^{{}^{\prime }}})}{R_{h^{{}^{\prime }}}X_{c^{{}^{\prime }}}-R_{c^{{}^{\prime }}}X_{h^{{}^{\prime }}}} \end{array}$$ (5)

$$\begin{array}{c} \tan(\theta_{1}+m\theta_{1})=\frac{Z_{1}(X_{h^{{}^{\prime }}}+X_{c^{{}^{\prime }}})}{R_{h^{{}^{\prime }}}R_{c^{{}^{\prime }}}+X_{h^{{}^{\prime }}}X_{c^{{}^{\prime }}}-Z_{1}^{2}} \end{array}$$ (6)

Solving for *Z*_1_ and *θ*_1_, the followings are obtained

$$\begin{array}{c} Z_{1}=\sqrt{\alpha R_{h^{{}^{\prime }}}^{2}+\frac{\left(\beta^{2}-\alpha \right)X_{h^{{}^{\prime }}}^{2}}{\alpha -1}},\theta_{1}=\frac{\pi +\tan^{-1}(\frac{Z_{1}\left(1-\alpha \right)}{X_{h^{{}^{\prime }}}\left(\beta -\alpha \right)})}{m+1} \end{array}$$ (7)

where *α* = *R*_*c*′_ / *R*_*h*′_ and *β* = *X*_*c*′_ / *X*_*h*′_.

*Y*_*in*,2_(*f*_*h*_) can be written as

$$\begin{array}{c} Y_{in,2}\left(f_{h}\right)=\frac{Z_{2}+jZ_{0}\tan\theta_{2}}{Z_{2}(Z_{0}+jZ_{2}\tan\theta_{2}).} \end{array}$$ (8)

To satisfy $Y_{in,2}(f_{h})=Y_{in,2}^{*}(f_{c})$ for real *Z*_2_ and *Z*_0_, tan(*θ*_2_) must be the negative value of tan(*mθ*_2_) from Eq (8). The result can be written as tan(*θ*_2_) = −tan(*mθ*_2_) = tan(−*mθ*_2_ − *π*), we conclude that *θ*_2_ = *π*/(*m* + 1). The condition *G*_1,*h*_ = *G*_2,*h*_ must be satisfied where *Y*_*in*,*n*_(*f*_*h*_) = *G*_*n*,*h*_ + *jB*_*n*,*h*_ and *Y*_*in*,*n*_(*f*_*c*_) = *G*_*n*,*c*_ + *jB*_*n*,*c*_. *G*_1,*h*_ = *G*_2,*h*_ can be written as $\left(Z_{2}Z_{0}+Z_{0}Z_{2}\tan^{2}\theta_{2}\right)/\left(Z_{2}\left(Z_{0}^{2}+Z_{2}^{2}\tan^{2}\theta_{2}\right)\right)=G_{1,h}$. By solving this equation, *Z*_2_ can be acquired, as follows:

$$\begin{array}{c} Z_{2}=\sqrt{\frac{Z_{0}\left(1-Z_{0}G_{1,h}+\tan^{2}\theta_{2}\right)}{G_{1,h}\tan^{2}\theta_{2}}}\;\text{where}\;\theta_{2}=\frac{\pi }{m+1}. \end{array}$$ (9)

The final step to realize impedance matching is to cancel susceptances (*B*_1,*h*_ + *B*_2,*h*_) and (*B*_1,*c*_ + *B*_2,*c*_) by using an open or shorted shunt stub. The susceptance of the stub must satisfy the following equation:

$$\begin{array}{c} j\left(B_{1,h}+B_{2,h}\right)=-jB_{3,h},\;\;j\left(B_{1,c}+B_{2,c}\right)=-jB_{3,c}. \end{array}$$ (10)

As the value of |*B*_1,*h*_ + *B*_2,*h*_| approaches zero, the characteristic impedance value of the single shunt stub becomes several thousand ohms; however, Nikravan and Atlasbaf demonstrated that using a single-section shunt stub under these conditions is problematic [6]. Alternatively, if we use a double-section stub, the characteristic impedances *Z*_*n*_(*n* = 3, 4) can be selected within the feasible impedance range. Using an open stub, *Y*_*in*,3_ for cold and hot states are respectively given as follows [8]:

$$\begin{array}{c} Y_{in,3}\left(f_{h}\right)=-j\frac{Z_{3}+Z_{4}\cot\theta_{4}\tan\theta_{3}}{Z_{3}\left(Z_{3}\tan\theta_{3}-Z_{4}\cot\theta_{4}\right)} \end{array}$$ (11)

$$\begin{array}{c} Y_{in,3}\left(f_{c}\right)=-j\frac{Z_{3}+Z_{4}\cot\left(m\theta_{4}\right)\tan\left(m\theta_{3}\right)}{Z_{3}\left(Z_{3}\tan\left(m\theta_{3}\right)-Z_{4}\cot\left(m\theta_{4}\right)\right)}. \end{array}$$ (12)

When *Z*_3_ and *Z*_4_ are determined, *θ*_3_ can be expressed as a function of *θ*_4_ from Eqs (11) and (12) as follows:

$$\begin{array}{c} \theta_{3}=\{\begin{array}{cc} \tan^{-1}(\frac{B_{3,h}Z_{3}Z_{4}\cot\theta_{4}+Z_{3}}{B_{3,h}Z_{3}^{2}-Z_{4}\cot\theta_{4}}), & \text{@}\;f_{h}\\ \frac{1}{m}\tan^{-1}(\frac{B_{3,c}Z_{3}Z_{4}\cot\left(m\theta_{4}\right)+Z_{3}}{B_{3,c}Z_{3}^{2}-Z_{4}\cot\left(m\theta_{4}\right)}), & \text{@}\;f_{c} \end{array} \end{array}$$ (13)

From Eq (13), we can determine *θ*_3_ and *θ*_4_ via numerical analysis. It should be noted that, for a shorted stub, *θ*_3_ and *θ*_4_ can be acquired using similar derivations.

### Verification of the proposed method

To validate a dual-state impedance matching method for plasma lamps, we have extracted the parameters of the dual-state matching circuit for the plasma lamp model (Table 1). The extracted impedance transformer component values corresponding to implementation of double-section open-circuited stub with *Z*_3_ = 60 Ω and *Z*_4_ = 10 Ω are listed in Table 2.

**Table 2:** Extracted component values of the dual impedance transformer.

Unit	*Z*_*T*_	*Z*_1_	*Z*_2_	*Z*_3_	*Z*_4_
Ω	50	10.24	32.4	60	10
Unit	*θ*_*T*_	*θ*_1_	*θ*_2_	*θ*_3_	*θ*_4_
degree	30.8	112.5	89.74	114.34	533.99

**Fig 4:**
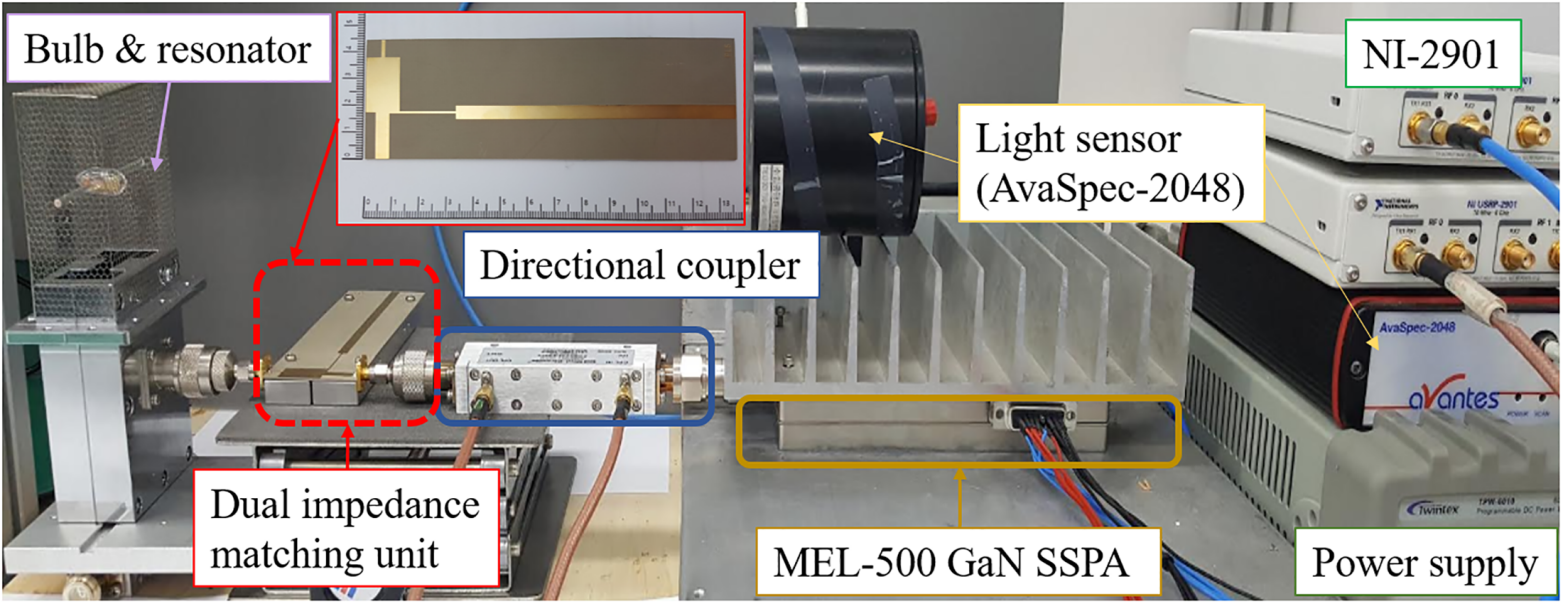
PLS test setup.

**Fig 5:**
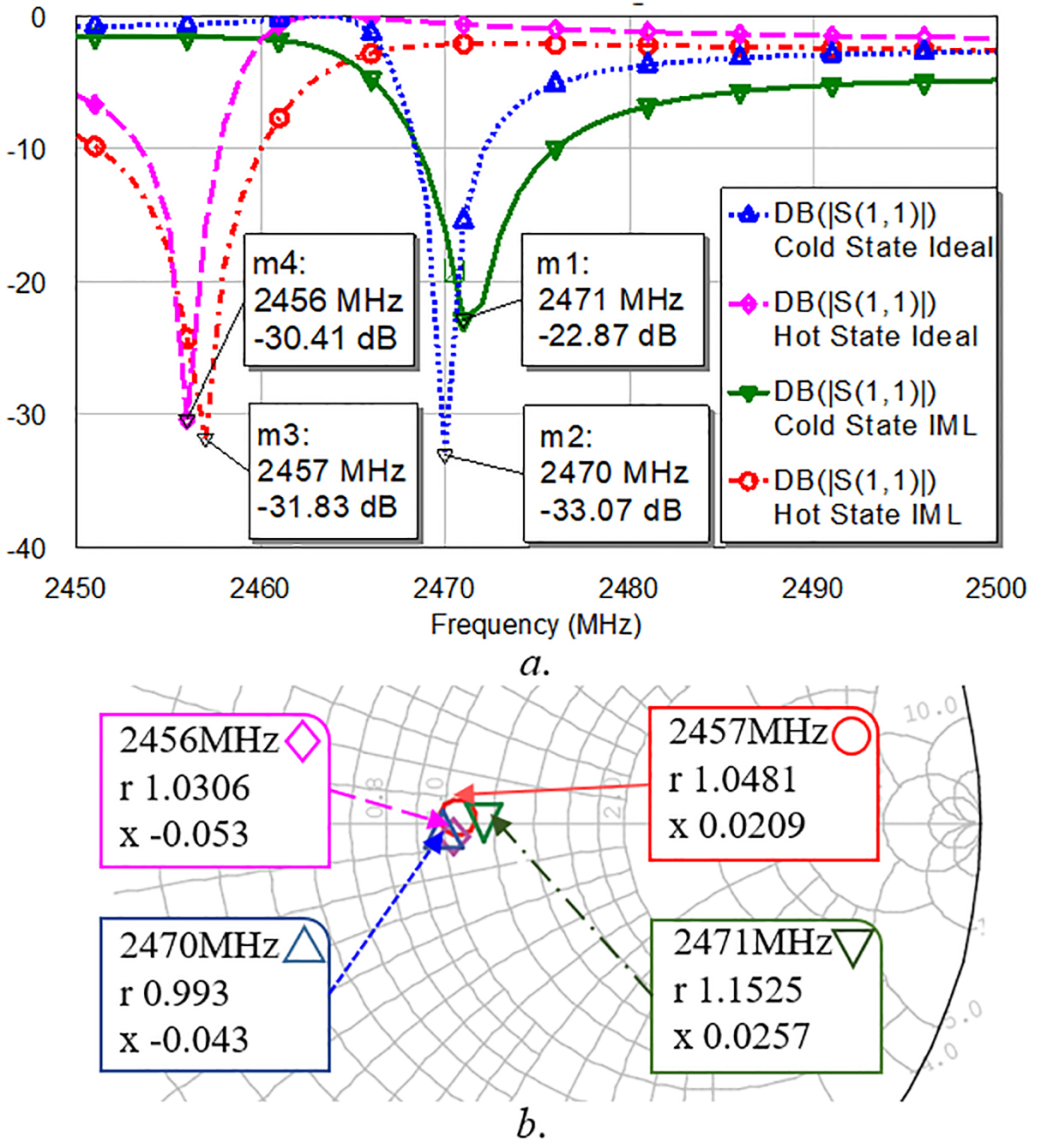
Return losses (Fig 5*a*) and impedances (Fig 5*b*) of cold and hot states following impedance transformer implementation.

The impedances of the plasma lamp with the dual-state impedance transformer were 50 Ω in the hot and the cold states at frequencies of 2456 and 2470 MHz, respectively. The dual impedance transformer is implemented using microstrip line (*ε*_*r*_ = 3.5, height = 0.706 mm Taconic substrate). The overall PLS test setup with the implemented microstrip line (IML) matching circuit is shown in Fig 4. Fig 5*a* and 5*b* show the simulated return losses and impedances of the load as resulting from ideal transmission line matching circuit and IML matching circuit respectively. The result of Smith chart in Fig 5*b* shows that the impedances for both cold and hot states are well matched for 50 Ω.

The results show a significant decrease in the return loss for both states at the given frequencies. By applying the proposed method, the return losses are simultaneously improved to -31 dB and -22 dB from -6 dB and -0.8 dB for hot and cold states, respectively. The proposed scheme allows dual-state impedance matching for two bands with a frequency spacing of 14 MHz, which is a narrower frequency interval than the previous results [4, 6–9]. Table 3 compares the performance of the proposed method with those of the previous methods. There are works of impedance matching for a plasma bulb [2, 4, 9], but they use a single matching for an impedance of one plasma state. The dual impedance matching methods [6–8] are not designed for a narrow frequency band gap because they do not consider very different impedances within a narrow frequency band gap. The proposed method is a new attempt to match two very different impedances in the near frequency band as far as the authors know.

**Table 3:** Comparison with the previous works.

	*f*_*h*_ [MHz]	*f*_*c*_/*f*_*h*_	|*f*_*c*_ − *f*_*h*_|	|*S*_11_|@*f*_*h*_	|*S*_11_|@*f*_*c*_	Matching
[2]	433	1.02	11.5 MHz	-	-2.6 dB	Single
[4]	2400	1.106	245 MHz	-10.4 dB	-32.8 dB	Single
[9]	2390	1.029	70 MHz	-40 dB	-10 dB	Single
[6]	6000	1.33	2000 MHz	-45 dB	-23 dB	Dual
[7]	1500	1.5	750 MHz	-23 dB	-35 dB	Dual
[8]	900	2.6	1500 MHz	-58 dB	-20 dB	Dual
This work	2457	1.005	14 MHz	-31 dB	-22 dB	Dual

The plasma lamp used in the experiment is turned on by contacting with a metal without the proposed dual matching. Using the proposed method, the plasma lamp can be turned on without metal contact and the low return loss in the hot-state can be achieved simultaneously.

## Conclusion

We propose an accurate plasma lamp circuit model and a corresponding dual-state impedance matching method that considers both cold and hot states. Subsequently, a plasma lighting system with 300 W GaN SSPA was implemented for model validation. Results showed that the dual matching between the SSPA and plasma lamp improved the power efficiency of the SSPA in the hot-state and facilitated bulb operation in the cold-state. Thus, the proposed model and matching method can be applied to improve the efficiency of an RF-driven energy system.

## Supporting information

S1 File Fig 2 Plasma bulb modeling and measurement. Comparison of measured and simulated impedances of plasma lamp.
(TXT)

S2 File Fig 5 Ideal cold and hot state S_11 data set compare with IML. (TXT)
